# Efficiency Enhancement of Solid-State CuInS_2_ Quantum Dot-Sensitized Solar Cells by Improving the Charge Recombination

**DOI:** 10.1186/s11671-019-2998-7

**Published:** 2019-06-06

**Authors:** Bowen Fu, Chong Deng, Lin Yang

**Affiliations:** 1 College of Physics Science and Technology, Hebei University, Baoding, 071002 China; 2 Key Laboratory of Semiconductor Photovoltaic Technology of Inner Mongolia Autonomous Region, School of Physical Science and Technology, Inner Mongolia University, Hohhot, 010021 China

**Keywords:** CuInS_2_ quantum dots, Solar cells, Charge recombination, Electron transfer

## Abstract

Copper indium sulfide quantum dots (CuInS_2_ QDs) were incorporated into a nanocrystalline TiO_2_ film by using spin coating-assisted successive ionic layer adsorption and reaction process to fabricate CuInS_2_ QD-sensitized TiO_2_ photoelectrodes for the solid-state quantum dot-sensitized solar cell (QDSSC) applications. The result shows that the photovoltaic performance of solar cell is extremely dependent on the number of cycles, which has an appreciable impact on the coverage ratio of CuInS_2_ on the surface of TiO_2_ and the density of surface defect states. In the following high-temperature annealing process, it is found that annealing TiO_2_/CuInS_2_ photoelectrode at a suitable temperature would be beneficial for decreasing the charge recombination and accelerating the charge transport. After annealing at 400 °C, a significantly enhanced photovoltaic properties of solid-state CuInS_2_ QDSSCs are obtained, achieving the power conversion efficiency (PCE) of 3.13%, along with an open-circuit voltage (V_OC_) of 0.68 V, a short-circuit photocurrent density (J_SC_) of 11.33 mA cm^−2^, and a fill factor (FF) of 0.41. The enhancement in the performance of solar cells is mainly ascribed to the suppression of charge recombination and the promotion of the electron transfer after annealing.

## Background

Owing to the merits of multiexciton generation and tunable band gap, quantum dot-sensitized solar cells (QDSSCs) have been considered as one of the ideal candidates for the new generation solar cells [1–4]. For the improvement of power conversion efficiency, it is essential to select a semiconductor material with the proper band gap. CuInS_2_ (CIS) is a direct band gap I-III-VI_2_ semiconductor compound with a near-optimal bulk band gap (1.5 eV) and has many advantageous features including the higher absorption coefficient (10^5^ cm^−1^), non-toxicity, and excellent stability [5–7]. To date, it has been demonstrated as a promising photosensitizer which has been successfully used in the field of QDSSCs [8–12].

The deposition process of QDs has a significant impact on the photovoltaic properties. As we have known, there are two major QD deposition approaches, i.e., the direct growth and post-synthesis assembly. Most of the researches are focused on the post-synthesis assembly method to fabricate solar cells [13–15]. For example, Wang et al. [16] controlled the Cu/In non-stoichiometric ratios of CIS QDs, achieving a PCE of 8.54%, which was a high efficiency for the CIS-based solar cells. Zhong’s group [17] explored an alloyed Zn-Cu-In-Se (ZCISe) QD sensitizer and deposited ZCISe and CdSe QDs on mesoporous TiO_2_, which achieved a PCE of 12.75%. However, this method suffers from the small loading amount of QDs and the disadvantageous status of electronic coupling between QD and TiO_2_. To increase the QD loading and enhance the ability of efficient electron transfer to TiO_2_, QDs could be directly grown on mesoporous TiO_2_ film by successive ionic layer adsorption and reaction (SILAR) [18–20]. Furthermore, developing a strategy to accelerate the charge transport and enhance the device stability could greatly enhance the photovoltaic performance and versatility of QD-sensitized TiO_2_ electrodes. It has been realized that the solid-state cell device architecture is desirable to retard the deterioration of long-term stability associated with liquid electrolytes [21, 22]. Despite the promise of solid-state cells, the efficiencies reported to date were lower. In the earlier reports, So and co-workers [23] fabricated a non-annealed heterojunction solar cell with a PCE of 1.16% by incorporating colloidal CIS nanocrystals into porous TiO_2_ network. Zhou et al. [24] introduced In_2_S_3_ buffer layer into the solar cell based on CuInS_2_, achieving a PCE of 1.06%. Chang et al. [25] developed the Cu_2_S-CuInS_2_-ZnS solid-state QDSSCs with a PCE of 2.52% through the SILAR process. The performance of such devices commonly gets worse due to the recombination between TiO_2_ and hole conductor, which is faster than the analogous process in the devices with liquid electrolyte. A significant approach used to decrease recombination and increase efficiency is to modify the QDs absorber or TiO_2_ photoanode, e.g., through increasing the loading amount of QDs, doping QDs to optimize the interfacial band alignment, or using of passivation layer.

In a previous study, we succeeded in fabricating solid-state devices using CuInS_2_ quantum dot-sensitized TiO_2_ photoanodes through SILAR method [26]. Herein, to further improve the efficiency of the device, we fabricated the solid-state solar cell by introducing CIS QDs into TiO_2_ mesoporous layer through spin coating-assisted SILAR process, completely filling QDs in the pores of TiO_2_ mesoporous layer. Through optimization of QD-sensitized TiO_2_ films by using the precise deposition based on SILAR, together with the annealing treatment for the photoelectrodes, the solar cell consequently exhibits a PCE of 3.13%. As far as we know, this result is one of the best performances of CIS-based solid-state QDSSCs.

## Methods

### Materials

Indium acetate (In(OAc)_3_, 99.99%) was purchased from Alfa Aesar. Copper(II) chloride dihydrate (CuCl_2_·2H_2_O, 99.99%), sodium sulfide nonahydrate (Na_2_S·9H_2_O, 99.9%), titanium isopropoxide (99.9%), hydrochloric acid (HCl, 37% in water), 2,2’,7,7’-tetrakis-(*N*,*N*-di-p-methoxyphenylamine)-9,9’-spirobifluorene (spiro-OMeTAD, 99.5%), chlorobenzene (anhydrous, 99.8%), 4-tert-Butylpyridine (tBP), bis(trifluoromethane)sulfonimide lithium salt (Li-TFSI), and acetonitrile (anhydrous, 99.8%) were purchased from Sigma-Aldrich. TiO_2_ paste (DSL 18NR-T) was obtained from Dyesol. All of the chemicals were utilized directly without further purification. Ultrapure deionized water was used for the preparation of aqueous solutions.

### Preparation

A TiO_2_ compact layer with a thickness of 70 nm was fabricated by spin-coating on the cleaned FTO glass at 4000 rpm for 30 seconds, using titanium isopropoxide (350 μL) and HCl (35 μL) diluted in ethanol (5 mL) as the precursor solution. The film was then annealed in the air starting at room temperature with 100 °C increments, holding for 10 min at each increment. At 500 °C, the film was annealed for an hour and then allowed to cool naturally. Next, the TiO_2_ mesoporous layer was fabricated by spin-coating the diluted 18NR-T paste on the compact layer at 800 rpm for 10 s, followed by a heat treatment to achieve a 2-μm thickness layer.

CIS QD-sensitized TiO_2_ thin film was prepared by spin coating-assisted SILAR. 80 μL of a mixture of 25 mM CuCl_2_ and 50 mM In(OAc)_3_ was dropped on the TiO_2_ mesoporous layer and then spin-coated at 800 rpm for 20 s. Subsequently, 80 μL of 100 mM Na_2_S was dropped and followed by spin-coating at 800 rpm for 20 s. The two steps were denoted as one cycle. Between each step, the film should be rinsed with deionized water and dried by N_2_. In order to enhance the crystallinity of CIS QDs, the photoelectrodes were annealed under nitrogen atmosphere at 200–500 °C for 30 min. Subsequently, the hole transport material (HTM) was spin-coated under N_2_ atmosphere by using a solution with a proper concentration of 300 mg of spiro-OMeTAD, 2.91 μL of chlorobenzene, 28.77 μL of tBP, and 126 μL of Li-TFSI. Finally, gold was deposited by thermal evaporation as a counter electrode and the active area of 0.09 cm^2^ was defined.

### Characterization

UV-vis absorption spectra were recorded on a UV-vis spectrophotometer (Perkin Elmer Lambda 950). Cross-sectional scanning electron microscopy (SEM) was characterized by FEI nova nano SEM450. The elemental mappings were characterized by an ORBIS energy dispersive spectroscopy (EDS), an accessory of the SEM. The current density-voltage (J-V) measurements for solar cells were carried out under the illumination of a solar simulator equipped with a 300 W xenon lamp (Model No. XES-100S1, SAN-EI, Japan) under the standard test conditions (25 °C, AM1.5, 100 mW·cm^-2^). The incident photon-to-current conversion efficiency (IPCE) was measured by an Enlitech QER3011 system equipped with a 150 W xenon light source. Electrochemical impedance spectroscopy (EIS) was carried out on an electrochemical workstation (Zahner, Zennium) under dark conditions at different forward biases from − 0.1 to − 0.5 V, applying a 20-mV AC sinusoidal signal over the constantly applied bias with the frequency ranging from 1 to 0.1 Hz. Time-resolved photoluminescence (TRPL) was employed by PL Spectrometer (Edinburgh Instruments, FLS 900), excited with a picosecond pulsed diode laser (EPL 445) at a wavelength of 543 nm.

## Results and Discussion

**Fig. 1:**
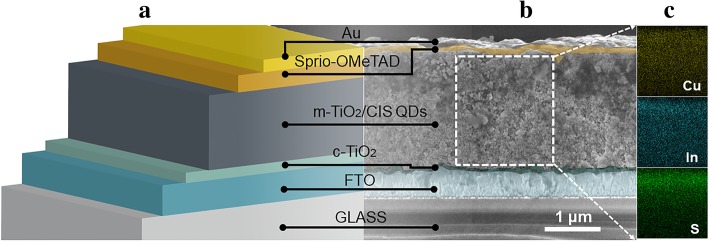
**a** Schematic of the device architecture. **b** The cross-sectional SEM image of the solar cell (corresponding to the sample prepared with 20 cycles and annealed at 400 °C). **c** The elemental distribution maps of Cu, In, and S elements in TiO_2_/CIS layer

A schematic of the device architecture is shown in Fig. 1, incorporating with the cross-sectional SEM image covered by false colors to distinguish the different layers prepared in the device. The uniform distribution of particles and superior contact between interfaces can improve the electrical conductivity of thin films that would enhance the charge carrier transfer [27–29]. The elemental mapping of CIS-sensitized TiO_2_ mesoporous film electrode is also performed through energy dispersive X-ray (EDX) analysis, providing clear evidence to prove the uniform distribution of CIS throughout the film.

**Fig. 2:**
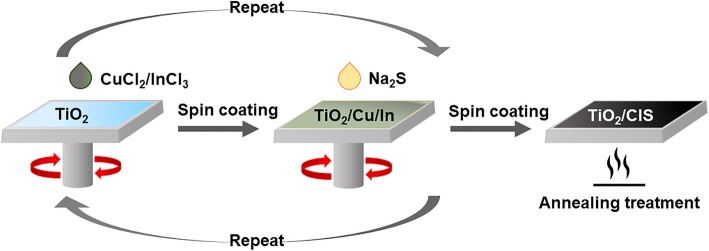
Schematic of the process for fabricating CIS QD-sensitized TiO_2_ photoelectrodes

**Fig. 3:**
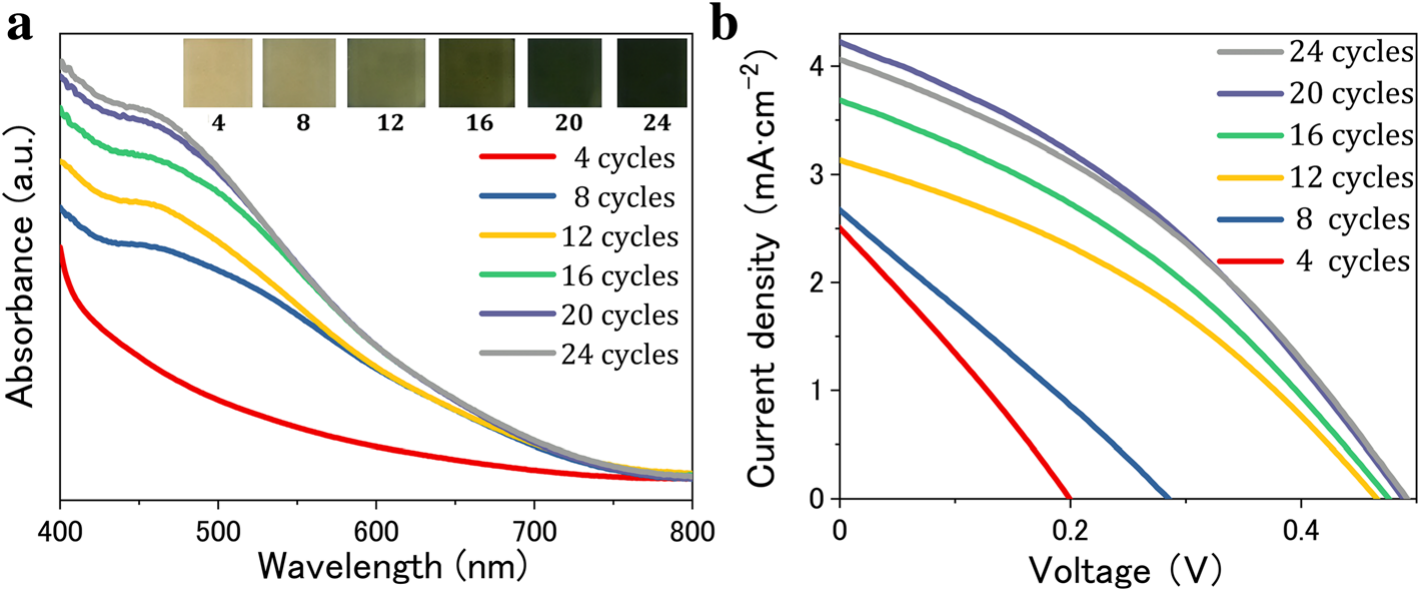
**a** UV-vis absorption spectra of CIS QD-sensitized TiO_2_ film prepared by spin coating-assisted SILAR with different cycles. The inset is the photographs of the corresponding photoelectrode films. **b** J-V curves of QDSSCs prepared at different cycles

The procedure of fabricating CIS QD-sensitized TiO_2_ photoelectrodes in our work is schematically illustrated in Fig. 2. It is worth pointing out that the spin coating-assisted SILAR method adopted in this work can control the amount of QD depositions accurately. The amount of CIS QDs incorporated in the mesoporous TiO_2_ layer was evaluated using the UV-vis absorption spectra. Figure 3a shows the variation of spectra with different spin coating-assisted SILAR cycles. After four cycles performed, only a much smaller amount of CIS QDs is deposited in TiO_2_ film, as indicated by the lower absorbance of TiO_2_/CIS photoelectrode. An increase in the number of cycles results in an increase of absorbance and a slight red shift of the absorption onset, corresponding to the color change of photoelectrodes from dark yellow to black, as shown in the inset of Fig. 3a. Subsequently, we fabricated and characterized the photovoltaic devices with TiO_2_/CIS photoelectrodes.

**Fig. 4:**
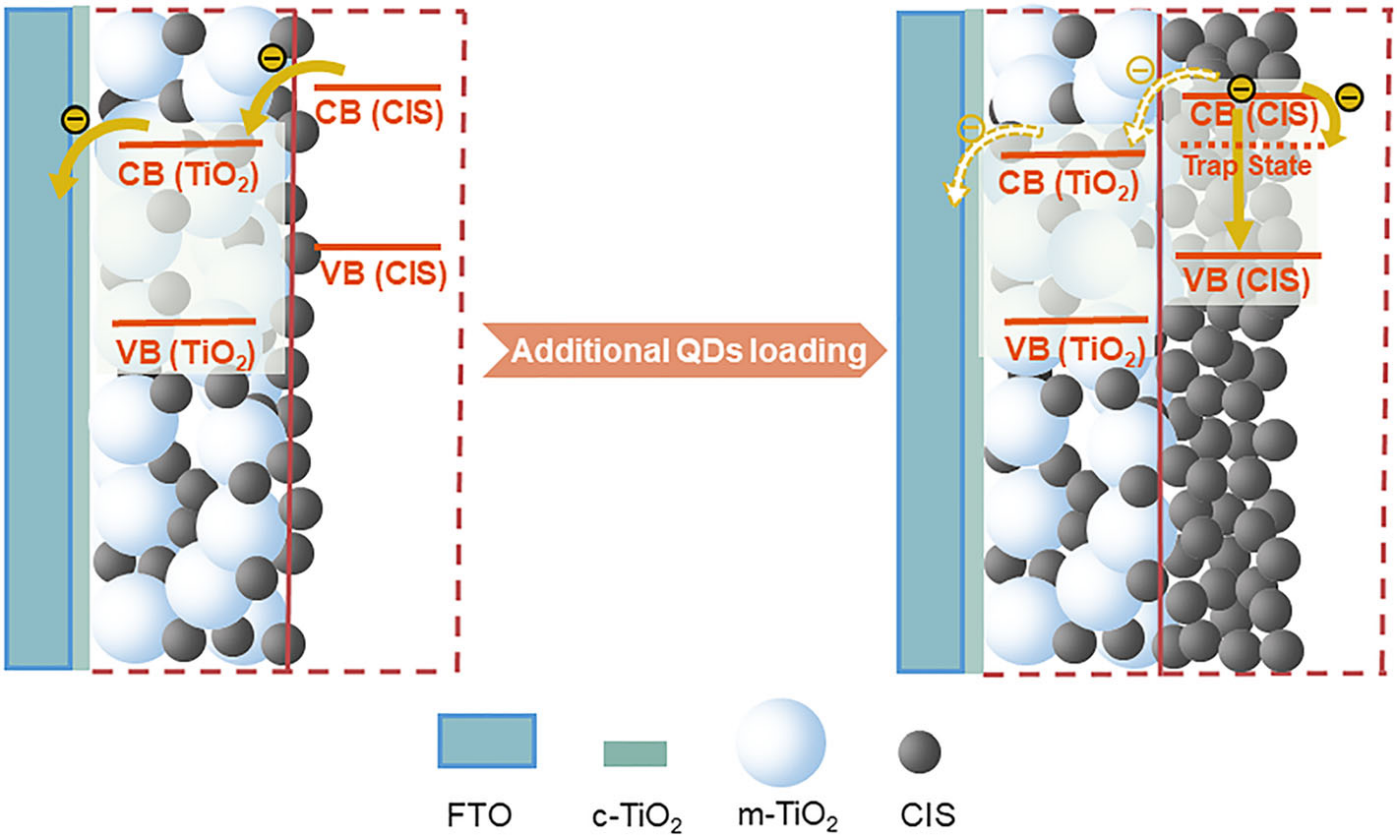
Schematic diagram of the major pathways of electron transfer and charge recombination existing in QDSSCs

Figure 3b shows the J-V curves of CIS QDSSCs. With increasing the number of spin coating-assisted SILAR cycles, both J_SC_ and PCE gradually increase from 2.49 mA cm^−2^ and 0.14% for 4 cycles to 4.21 mA cm^−2^ and 0.75% for 20 cycles, and then decrease to 4.05 mA cm^−2^ and 0.72% for 24 cycles, respectively, as clearly revealed in Table 1. This result demonstrates that the cycle process in the initial stage aims to increase the coverage of CIS QDs through refilling the uncovered areas in TiO_2_ mesoporous layer. It is no doubt that an enhancement of QD loading amount and a formation of QDs monolayer on the surface of TiO_2_ photoanode are advantageous to generate much more excited electrons under light illumination, which would increase the photocurrent of solar cells [30]. Moreover, a higher surface coverage for TiO_2_ is achieved with the increase of CIS QDs loading amount. The decrease of surface areas exposed directly to the HTM is favorable for the suppression of charge recombination process occurring at TiO_2_/HTM interface, thus leading to a dramatic increase of V_OC_ and an improvement of FF, especially in the early cycles. However, the thickness of the CIS layer could continuously increase after each spin coating-assisted SILAR cycle owing to the additional QD loadings. Because of the increased generation probability of charge recombination in CIS layer, the process of transporting photogenerated electrons from the QD layers to the TiO_2_ matrix will become more difficult, as shown in the schematic drawing of Fig. 4. The electrons in the QD conduction band (CB) can be trapped by the surface defect states [31, 32], which serve as the recombination centers, eventually giving a deterioration of the device. Meanwhile, the undesirable recombination path of the electrons in QD CB and the holes in QD VB could hinder the electron injection from CIS into TiO_2_ as well. Therefore, after the evaluation and verification of these effects, it clearly indicates that the ideal number of cycles (20) should be performed for the deposition CIS QDs in this work.

**Table 1:** Photovoltaic parameters obtained from the J-V curves of QDSSCs using TiO_2_/CIS films prepared with different cycles as photoelectrodes

Cycles	V_OC_ (V)	J_SC_ (mA cm^−2^)	FF	PCE (%)
4	0.20	2.49	0.28	0.14
8	0.28	2.65	0.26	0.20
12	0.46	3.12	0.36	0.51
16	0.47	3.68	0.36	0.62
20	0.48	4.21	0.37	0.75
24	0.49	4.05	0.36	0.72

**Fig. 5:**
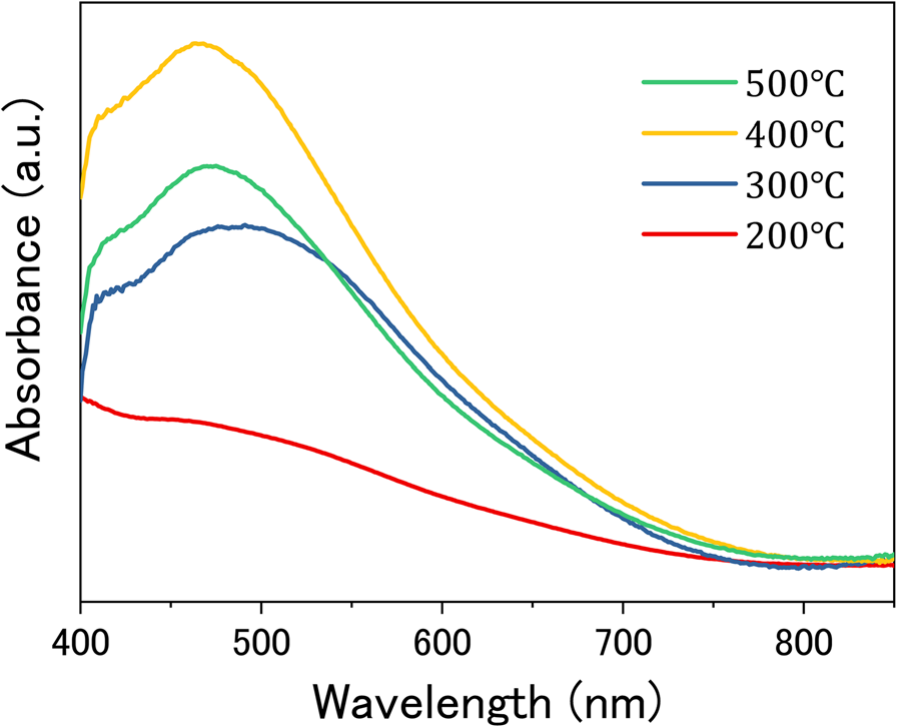
UV-vis absorption spectra of TiO_2_/CIS photoelectrodes with annealing treatment at different temperatures

Afterward, the influence of annealing treatment on the performance of photovoltaic devices is evaluated. Figure 5 presents the evolution of the absorption of CIS QD-sensitized TiO_2_ films with different annealing temperatures. It is found that the absorption is improved gradually with increasing the annealing temperature. The absorbance gets to a saturation value at the temperature of 400 °C. Simultaneously, excessive annealing treatment would deteriorate CIS QD sensitizer due to the occurrence of aggregation and oxidization [33]. It results in a decrease in absorbance when the annealing temperature is further elevated to 500 °C. Therefore, it is inferred that an excessive increase of annealing temperature (> 400 °C) is disadvantageous to the performance of cell devices.

**Fig. 6:**
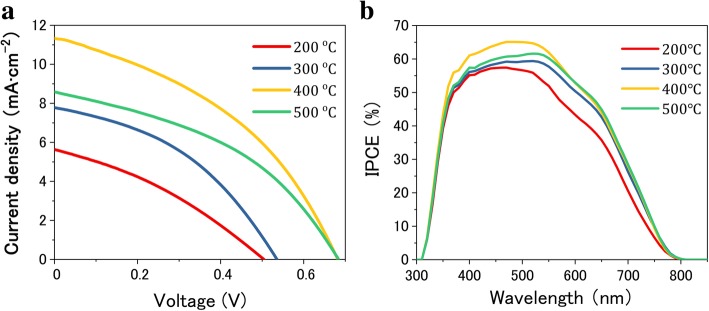
**a** J-V curves and **b** IPCE spectra of the cell devices based on TiO_2_/CIS photoelectrodes with annealing treatment at different temperatures

The J-V curves of QDSSCs which were measured under simulated AM1.5 sunlight illumination are shown in Fig. 6a, comparing the photocurrent-photovoltage characteristics of cell devices with different annealing temperatures. The detailed parameters are listed in Table 2. The device based on the photoelectrode annealed at 200 °C shows a much lower J_SC_ of 5.63 mA cm^−2^. A relatively higher J_SC_ of 7.76 mA cm^−2^ was obtained by annealing the TiO_2_/CIS photoelectrode at 300 °C. At 400 °C, the device exhibits the highest PCE of 3.13%, along with V_OC_ of 0.68 V, J_SC_ of 11.33 mA cm^−2^, and FF of 0.41. The enhanced J_SC_ results from the beneficial light-harvesting enhancement over the UV-vis spectrum for the photoelectrodes with annealing treatment at a higher temperature. Nevertheless, with increasing the temperature up to 500 °C, it is no longer able to bring an improvement in the performance of solar cells, unfortunately causing a significant decline in J_SC_ and PCE. So the film annealed at 400 ^°^C exhibits the best photovoltaic performance as compared with the other three samples. To assess the light absorption and the electron generation characteristics, IPCE spectra are shown in Fig. 6b. It exhibits a strong photoresponse with a value of 66% in the visible wavelength range between 400 and 550 nm for QDSSCs with the annealing temperature of 400 ^°^C, with nearly 20% enhancement compared to that of 200 ^°^C. The higher IPCE response generally ascribed the outstanding absorptivity of QDs in the spectral region. According to the spectral, it can be found that a wider response wavelength range and a higher IPCE value appeared, which is in accordance with the variation tendency of J_SC_ as observed in J-V measurement. The result could be supported by the interpretation that the proper annealing treatment is potentially more favorable for the formation of an enforced interface connection between CIS and TiO_2_, thus leading to the effective electron transfer in QDSSCs [34].

**Table 2:** Photovoltaic parameters obtained from the J-V curves of QDSSCs using TiO_2_/CIS films annealed at different temperatures as photoelectrodes

Temperatures	V_OC_ (V)	J_SC_ (mA cm^−2^)	FF	PCE (%)
200 °C	0.50	5.63	0.37	1.04
300 °C	0.53	7.76	0.41	1.68
400 °C	0.68	11.33	0.41	3.13
500 °C	0.68	8.58	0.42	2.44

**Fig. 7:**
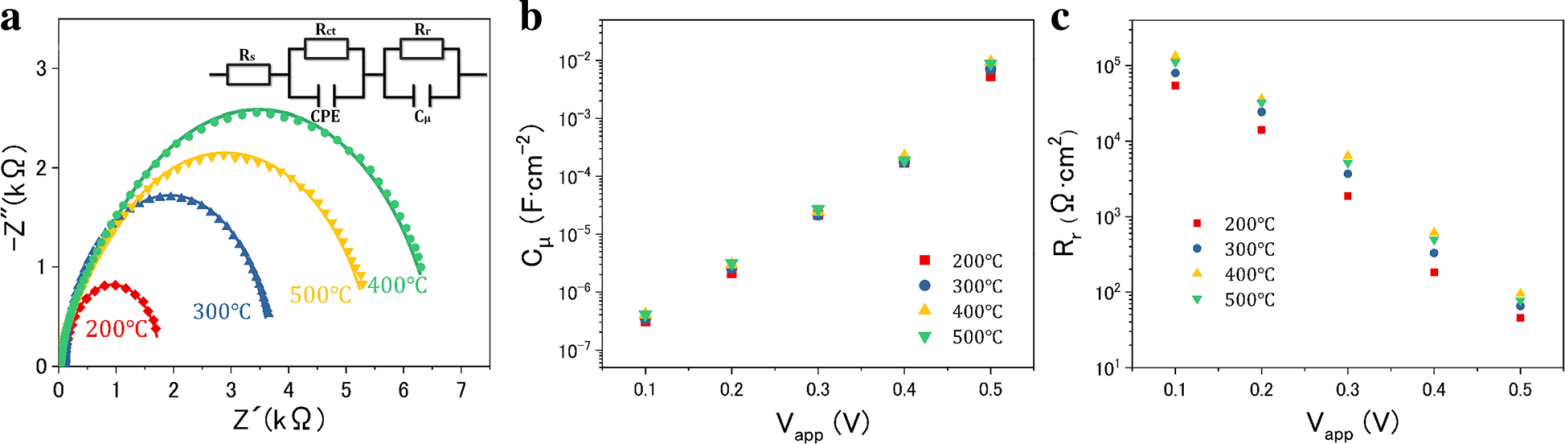
**a** EIS spectra of the cell devices measured in the dark at − 0.4 V bias. The inset in **a** illustrates the equivalent circuit simulated to fit the impedance spectra. R_S_ represents the substrate resistance. R_ct_ and CPE represent the charge transfer resistance and capacitance at the HTM/counter electrode interface, respectively. *R*_r_ and *C*_μ_ represent the recombination resistance and chemical capacitance at the photoelectrode/HTM interface, respectively. **b** *C*_μ_ and **c** *R*_r_ at different applied voltages (V_app_), calculated from the fitting of the impedance spectra

To analyze the transfer and recombination process of charge carrier, the devices are further investigated by EIS. Figure 7a displays the Nyquist plot of the obtained EIS results at − 0.4 V bias, and the fitted values evaluated from the equivalent circuit are listed in Table 3, where the electron lifetime can be estimated by *τ*_n_ = *R*_r_ × *C*_μ_ [35–37]. At the HTM/counter electrode interface, the charge transfer resistance R_ct_ which is related to the high-frequency semicircles presents no obvious differences, while the same HTM and counter electrode were employed in the present QDSSCs. The simulated datum of recombination resistance *R*_r_ which is related to the low-frequency semicircles represents the electron transfer process at photoelectrode/HTM interface. This datum for QDSSCs with TiO_2_/CIS photoelectrode annealed at 400 °C is larger as compared with the others, which is attributed to the suppressed interfacial recombination, resulting in an enhanced V_OC_. Furthermore, the long-lived charge carriers could favor the improvement of charge collection efficiency, thereby contributing to the significant progress in IPCE and J_SC_ [6]. According to Table 3, in the present case, the TiO_2_/CIS photoelectrode annealed at 400 °C is indicated to remain the highest value of *τ*_n_, ∼ 117 ms, thus yielding the highest value of J_SC_ as observed in the J-V measurement. Nevertheless, *τ*_n_ falls to ∼ 78 ms when the higher temperature of 500 °C was applied. The V_app_-dependent *C*_μ_ and *R*_r_ extracted from EIS measurements are illustrated in Fig. 7b and c, respectively. *C*_μ_ increases exponentially with the V_app_, as expected from the theoretical basis. The similar *C*_μ_ values of all the cells illustrate that different annealing temperatures do not produce a shift on the position of TiO_2_ CB [38, 39]. In addition, with increasing the temperature from 200 to 400 °C, the *R*_r_ value is improved gradually. Since the recombination rate occurring at photoelectrode/HTM interface is inversely proportional to *R*_r_ [39], the greater value of *R*_r_ means the reduced recombination rate occurring in the solar cell based on TiO_2_/CIS photoelectrode annealed at 400 °C. Overall, from these EIS results, it can be concluded that the cell devices show a large recombination rate rather than a shift of TiO_2_ CB. It also supports the lower recombination rate and longer electron lifetime for the solar cell based on TiO_2_/CIS photoelectrode annealed at 400 °C, which is conducive to the enhanced V_OC_, J_SC_, and FF values for cells undergoing annealing treatment on photoelectrodes as observed in the J-V curves.

**Table 3:** Fitted values of resistance, capacitance, and electron lifetime in EIS spectra at − 0.4 V bias

Temperatures	R_s_ (Ω)	R_ct_ (Ω cm^2^)	CPE (mF/cm^2^)	*R*_r_ (Ω·cm^2^)	*C*_μ_ (mF/cm^2^)	*τ*_n_ (ms)
200 °C	67.87	11.88	0.40	153	0.12	18
300 °C	65.58	17.17	0.50	329	0.14	46
400 °C	60.68	16.66	0.40	618	0.19	117
500 °C	64.36	15.68	0.44	495	0.16	79

**Fig. 8:**
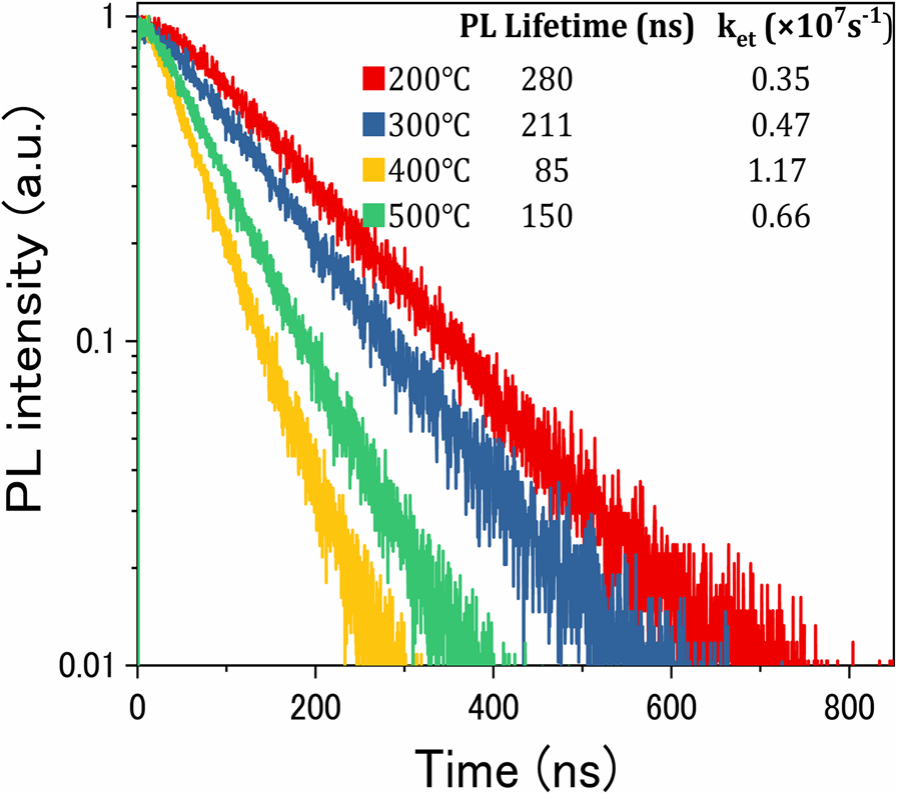
TRPL spectra of CIS QD-sensitized TiO_2_ films. The inset presents the PL lifetime and the rate of electron transfer

In order to further clarify the effect of annealing temperature on charge transfer, the time-resolved transient photoluminescence (TRPL) spectra of the samples are displayed in Fig. 8. It can be seen that the PL lifetime of the photoanode significantly decreases with the increase of the annealing temperature, which indicates that more electrons could transfer from CIS to TiO_2_ efficiently, reducing the probability of internal photogenerated carrier recombination inside QDs to some extent. According to the calculation of the rate of electron transfer (k_et_) [40, 41], it can be observed that the solar cell based on TiO_2_/CIS photoelectrode annealed at 400 °C has the higher k_et_ value of 1.17 × 10^7^ s^−1^, thus providing excellent charge transfer performance of QDSSCs. Consequently, it provides further evidence to support that the proper annealing treatment is potentially more favorable for obtaining an effective connection at TiO_2_/QDs interfaces [33], which is extremely beneficial for the transport of charge carrier in QDSSCs, thereby leading to a higher efficiency.

## Conclusions

In summary, CIS QD-sensitized TiO_2_ films were obtained by the spin coating-assisted SILAR method and further used as the promising photoelectrodes for solid-state QDSSCs. The spin coating-assisted SILAR method can control the amount of QD deposition accurately. Increasing the number of cycles could enhance the absorption ability, leading to more electrons generated under light illumination. The charge recombination process occurring at TiO_2_/HTM interface would be suppressed with the increase in the QD loading amount as well. However, there would appear the undesirable recombination pathways in the thicker CIS layer due to the excessive increase in the number of cycles, which is extremely detrimental to the device performance. The following high-temperature annealing treatment plays a critical role in enhancing the contact between CIS QDs and TiO_2_ photoanode and reducing the probability of internal photogenerated carrier recombination. According to J-V characteristics and EIS results, the most suitable annealing temperature for TiO_2_/CIS photoelectrode film should be 400 °C, which shows the highest efficiency of 3.13% and the longest electron lifetime of 117 ms. IPCE of 66% between 400 and 550 nm and k_et_ of 1.17 × 10^7^ s^−1^ are also achieved with the solid-state QDSSCs. This work may enlighten the way to fabricate the other kinds of sensitized photoelectrodes with high photovoltaic performance, and the next work will focus on improving the stability of cell devices.

## Abbreviations

CB: Conduction band
CIS QDs: Copper indium sulfide quantum dots
EDS: Energy dispersive spectroscopy
EIS: Electrochemical impedance spectroscopy
FF: Fill factor
IPCE: Incident photon-to-current conversion efficiency
J_SC_: Short-circuit photocurrent density
PCE: Power conversion efficiency
QDSSCs: Quantum dot-sensitized solar cells
SEM: Scanning electron microscopy
SILAR: Successive ionic layer adsorption and reaction
TRPL: Time-resolved photoluminescence
VB: Valence band
V_OC_: Open-circuit voltage
